# The co-occurrence of non-suicidal self-injury and attempted suicide among adolescents: distinguishing risk factors and psychosocial correlates

**DOI:** 10.1186/1753-2000-6-11

**Published:** 2012-03-30

**Authors:** Margaret S Andover, Blair W Morris, Abigail Wren, Margaux E Bruzzese

**Affiliations:** 1 Department of Psychology, Fordham University, 441 East Fordham Road, Bronx, NY 10458, USA

**Keywords:** Non-suicidal self-injury, Suicide attempt, Deliberate self-harm, Adolescence

## Abstract

Although attempted suicide and non-suicidal self-injury (NSSI) are distinct behaviors differing in intent, form, and function, the behaviors co-occur at a high rate in both adults and adolescents. Researchers have begun to investigate the association between attempted suicide and NSSI among adolescents. The purpose of this paper is to present current research on this association. First, we discuss definitional issues associated with self-injurious behaviors. Next, we present research on the co-occurrence of attempted suicide and NSSI, including prevalence and associations with self-injury characteristics. We then discuss psychosocial variables associated with engaging in both NSSI and attempted suicide or one type of self-injury alone. Finally, we present the research to date on risk factors uniquely associated with either attempted suicide or NSSI. Implications for mental health professionals and future avenues of research are discussed.

## Background

Suicide and non-suicidal self-injury (NSSI) are major public health concerns among adolescents [1]. In the United States, suicide is the 3rd leading cause of death among adolescents and young adults, and the 17th leading cause of death for children aged 5 to 9 years [2]. Attempted suicide is common in both clinical and community samples; 6.3% of high school students report at least one suicide attempt in the past year [3]. This statistic is especially concerning given that a prior history of attempted suicide is a significant predictor of future suicide attempts and suicide death [e.g., [4]]. NSSI is also surprisingly prevalent among community samples of adolescents, with rates ranging from 13 to 46.5% [5-7]. The behavior generally onsets between 12 and 14 years of age [1], making adolescence an important target of research and clinical attention.

A significant number of adolescents report engaging in both attempted suicide and NSSI [8-13]; this finding has stimulated a growing body of research on the association between NSSI and attempted suicide and the factors that differentiate the two types of self-injury. The purpose of this paper is to present an overview of the association between NSSI and attempted suicide among adolescents and the risk factors that differentiate the behaviors. As such, our goals are threefold. First, we will review the definitional issues associated with self-injurious behaviors with and without suicidal intent. Second, we will review research on the co-occurrence of NSSI and attempted suicide among adolescents, including prevalence and clinical characteristics of the behaviors and their co-occurrence. Finally, we will review empirical research on the factors uniquely associated with self-injury with or without suicidal intent among adolescents.

### Definitional issues

Both involving deliberate injury to the body, suicide and NSSI are differentiated by the presence or absence of suicidal intent. Despite this accepted distinction, the field of self-injury research has been plagued with definitional challenges. Limitations in the measurement of suicide and NSSI exist due in part to a lack of standardized nomenclature and clear operational definitions [14]. Despite numerous attempts to develop a consistent system of classification for self-injurious behaviors [e.g., [15-18]], a single system has not yet been widely accepted. Part of the difficulty in establishing standard terminology rests in the ambiguity of the constructs themselves. By definition, suicidal intent is used to distinguish between NSSI and suicide. However, suicidal intent may be ambiguous; this can be reflected in the ambivalence towards death sometimes reported by individuals engaging in attempted suicide and NSSI [19]. For example, any self-injurious act performed with a level of suicidal intent-even if suicidal intent is uncertain-is categorized as a suicide attempt [e.g., [14]. Although this is consistent with the definition of NSSI, it results in further heterogeneity among individuals who have attempted suicide. In addition, certain behaviors with apparent suicidal intent, such as overdoses and self-poisonings, are not considered to be methods of NSSI, regardless of self-reported intent to die [20].

A result of the lack of standard nomenclature is that different terms are sometimes used interchangeably to reference a single concept, while a single term can be used to reference several different concepts [17]. One of the most salient examples of this is in the use of the terms deliberate self-harm (DSH) and NSSI. The term DSH can be used to include all purposeful self-injury or self-poisoning episodes regardless of suicidal intent [e.g., [21]], or to represent non-fatal self-injury lacking suicidal intent [e.g., [22]], also referred to as NSSI. Use of the same term to describe different behaviors-and use of different terms to describe the same behavior-creates significant challenges when seeking to compare research findings. For the purposes of this paper, a suicide attempt is defined as a purposeful self-inflicted non-fatal injury performed with intent to die [18], and NSSI is defined as deliberate, self-inflicted destruction of body tissue without suicidal intent and for purposes not socially sanctioned.

### Co-occurrence of NSSI and suicide

Although suicide and NSSI both involve deliberate tissue damage, the behaviors are phenomenologically distinct. As discussed above, the most basic distinction between the behaviors is suicidal intent, as NSSI is performed without intent to die, and suicide attempts are performed with at least some intent to die. Muehlenkamp [23] outlined additional characteristics differentiating NSSI from attempted suicide. Suicide attempts are generally associated with thoughts of death and dying, while NSSI is generally associated with an intent to alleviate distress. Suicide attempts tend to occur with low frequency, a single method, and high lethality injuries, whereas NSSI tends to occur chronically, with high frequency, multiple methods, and low lethality injuries. With regards to response from the environment, suicide attempts often elicit reactions of care, compassion, and concern; in contrast, NSSI often elicits responses involving disgust, fear, and hostility. Unlike suicide attempts, NSSI results in calm and relief, even satisfaction, upon completion [23]. The behaviors also generally differ in function; adolescents with a history of attempted suicide are significantly more likely than those with an NSSI history to report self-injuring to stop negative thoughts or to "see if anyone loves [them]" (p. 62) [24]. Characteristics of self-injury thoughts also differ based on type of self-injury. Among adolescents with histories of both NSSI and attempted suicide, most had one thought of NSSI per day that was of moderate intensity and lasted less than 30 minutes. However, suicidal thoughts were longer, occurred less frequently, and were less likely to lead to self-injurious behaviors than thoughts of NSSI [25].

Despite the differences between NSSI and attempted suicide, a significant number of adults and adolescents report a history of both behaviors. Among clinical samples of adolescents, 14-70% report histories of both NSSI and attempted suicide [8-13]. Among adolescents with treatment-resistant depression, approximately twice as many youth with a history of one form of self-injury (either NSSI or attempted suicide) reported a history of the other form than those without the index form of self-injury, further supporting the co-occurrence of the behaviors [8]. Co-occurring NSSI and attempted suicide is also evident among nonclinical samples; 3.8% to 7% of high school students report a history of both behaviors [6,26]. In addition, the co-occurrence of self-injury is not limited to behaviors; thoughts of NSSI frequently co-occur with thoughts of suicide. Over 40% of adolescents receiving emergency crisis services reported thoughts of suicide accompanied by thoughts of NSSI in the past 24 hours [27].

Researchers have begun to investigate the overlap between NSSI and attempted suicide beyond prevalence and co-occurrence of the behaviors among adolescents. Findings suggest that engagement in one type of self-injury (i.e., attempted suicide or NSSI) may be associated with engagement in and characteristics of the other type of self-injury. For example, among adolescents with histories of both NSSI and attempted suicide, Nock and colleagues [13] found that as the number of lifetime suicide attempts increased, the number of NSSI methods used and the number of years engaging in NSSI also increased. Although number of NSSI episodes was not associated with number of suicide attempts in a sample of adolescent psychiatric inpatients [13], adolescents with histories of both NSSI and attempted suicide engage in significantly more episodes of self-injury than adolescents with a history of one type of self-injury alone [12]. Although additional research is necessary to understand the characteristics of overlapping self-injury, these findings suggest that engagement in both NSSI and attempted suicide may be associated with specific self-injury characteristics than engagement in only one type of self-injury.

The co-occurrence between suicide attempts and NSSI may be explained in part by the interpersonal-psychological theory of attempted and completed suicide. Although this is not the only theory of attempted suicide or NSSI that could be applied to understanding the behaviors, the interpersonal-psychological theory has direct and specific implications for the mechanisms underlying the association between NSSI and attempted suicide. According to Joiner and colleagues [28,29], lethal suicidal behavior requires both the desire to die by suicide (influenced by perceptions of burdensomeness and thwarted belongingness) and ability to carry out lethal self-injury. Of particular importance to understanding the association between NSSI and attempted suicide is the ability to self-injure. It is theorized that this ability is acquired through habituation to physical pain, emotional pain, and fear, which takes place through repeated exposure to activities such as repeated suicide attempts, risk-taking behaviors, and vicarious exposure to such behaviors [29]. In addition, NSSI may habituate an individual to physical and emotional pain and the act of self-injury itself, thereby increasing future risk for death by suicide [28,29]. This theory provides one framework for understanding the co-occurrence of both NSSI and attempted suicide, as well as the consequences of compounded self-injury.

### Risk factors for NSSI and attempted suicide among adolescents

Given the gravity of NSSI and attempted suicide and the prevalence of the behaviors among adolescents, it is important to understand the factors that increase risk for self-injury, as well as factors that may be associated with increased severity or impairment. In addition, to better understand the relations between attempted suicide and NSSI, researchers must investigate risk factors and psychosocial variables associated with both behaviors and differentially associated with one behavior or the other. Significant research has been devoted to identifying risk factors for attempted suicide among adolescents [e.g., [4,30-32]], and researchers have begun to identify factors that increase NSSI risk [e.g., [1,33]]. However, few studies have investigated factors that are differentially associated with specific types of self-injury among adolescents. The majority of studies that inform our understanding of the association between NSSI and attempted suicide have investigated psychosocial factors among adolescents who report both NSSI and attempted suicide and adolescents who report a history of only one type of self-injurious behavior.

#### Factors associated with both NSSI and attempted suicide

Generally, individuals with a history of both types of self-injurious behaviors demonstrate increased symptomology in comparison with individuals with NSSI or attempted suicide alone (See Table 1). For example, researchers have investigated the associations among types of self-injury and specific psychiatric diagnoses. Guertin and colleagues [11] found that adolescents with a history of both NSSI and attempted suicide were more likely to meet diagnostic criteria for oppositional defiant disorder, major depressive disorder (MDD), and dysthymia than adolescents with a history of attempted suicide alone. However, Jacobson and colleagues [12] reported no difference in rates of MDD and post-traumatic stress disorder (PTSD) between adolescents with a history of both types of self-injury and those with a history of attempted suicide alone, although both groups were more likely to be diagnosed with MDD and PTSD than adolescents with a history of NSSI alone. Other research has investigated psychiatric symptoms rather than diagnoses among adolescents with histories of self-injurious behaviors. Adolescents with a history of NSSI and attempted suicide report more symptoms of depression [12,34] and anhedonia [6,26] than those with a history of NSSI alone, although Brausch and Gutierrez [26] found that the groups did not differ in dysphoric mood or somatic symptoms of depression. Adolescents with histories of both types of self-injury reported more symptoms of borderline personality disorder (BPD), including confusion about self, impulsivity, emotion dysregulation, than those with a history of NSSI alone; reported symptoms did not differ significantly between those with NSSI alone and those with a suicide attempt alone [35]. In addition, adolescents with histories of NSSI and attempted suicide report greater hopelessness, loneliness, anger, risk taking, reckless behaviors, and alcohol use than those with histories of attempted suicide alone [11]. Compared with adolescents with an NSSI history alone, those with a history of both NSSI and attempted suicide report more negative self-evaluation [6], lower self-esteem [26], and greater impulsivity [34].

**Table 1:** Clinical factors significantly associated with self-injury with and/or without suicidal ideation

Factors associated with NSSI + SA	Factors associated with NSSI	Factors associated with SA
Depressive symptoms [11,12,34]	Less suicidal ideation [12]	Depressive symptoms [12,36]
Suicidal ideation [6,26,34]	Interpersonal chaos [35]	More stressful life events [24,36]
Fewer reasons for living [6]	Physical abuse [9]	Exposure to a suicide attempt [24,36]
Anhedonia [6,26]	Any maltreatment [9]	Suicidal ideation [36]
Negative self-evaluation [6,26]		Anxious symptoms [36]
Impulsivity [34]		Substance use [36]
Hopelessness [34]		MDD [12]
Loneliness [11]		PTSD [12]
Anger [11]		Confusion about self [35]
Confusion about self [35]		Fewer family relationships [36]
Less self-esteem [26]		Physical abuse [24]
Risk taking [11]		Death of a friend [24]
Reckless behavior [11]		Worries about sexual identity [24]
ODD [11]		
MDD [11,12]		
Dysthymia [11]		
Alcohol use [11]		
PTSD [12]		
Sexual abuse [9]		
Physical abuse [9]		
Any maltreatment [9]		
Less parental support [26]		

*Note*. NSSI = Non-suicidal self-injury. SA = Suicide attempt. NSSI + SA = histories of both NSSI and SA. ODD = Oppositional defiant disorder. MDD = Major depressive disorder. PTSD = Post-traumatic stress disorder

Although research suggests that adolescents with a history of both NSSI and attempted suicide may be more clinically severe than those with one type of self-injury alone, the finding of increased severity among adolescents with histories of both types of self-injury may not extend to all psychological constructs. For example, Brausch and Gutierrez [26] found no differences in body dissatisfaction or disordered eating among those with a history of both NSSI and attempted suicide or NSSI alone. Experiences of childhood adversity also did not differ between adolescents with both NSSI or attempted suicide or one type. Boxer [9] found that adolescents with a history of NSSI alone were as likely as adolescents with a history of NSSI and attempted suicide to report any type of childhood maltreatment, physical abuse, emotional abuse, and neglect, suggesting that childhood abuse and neglect may be a risk factor for self-injury in general. However, level of parental support may differentiate adolescents with a history of both NSSI and attempted suicide from those with an NSSI history alone; adolescents with a history of NSSI and attempted suicide reported less parental support than those with NSSI alone, but the groups did not differ in reported peer support [26]. Overall, research suggests that adolescents with histories of both NSSI and attempted suicide demonstrate more severe psychiatric symptomology and engage in more dangerous, risk taking behaviors than adolescents who engage in only one type of self-injury, highlighting the importance of assessing for both suicide and NSSI in clinical practice, as well as the need for intervention in this group.

#### Factors associated with NSSI or attempted suicide

Much of the research on the association between NSSI and attempted suicide has compared adolescents who have engaged in both types of self-injury to those who have engaged in only one type or who have never engaged in self-injury. However, comparing adolescents with only one type of self-injury to those with both limits our ability to determine unique risk factors for and predictors of attempted suicide and NSSI separately. Research suggests that history of a suicide attempt may be associated with more severe symptoms than NSSI. Adolescents with a suicide attempt history report greater levels of anxious and depressive symptoms [36] and more stressful life events [24,36] than those with an NSSI history. Adolescents with a suicide attempt history were also more likely to report physical abuse [24], substance use, and fewer family relationships [36]. Adolescents with suicide attempt histories and NSSI histories report similarly high levels of conflict with friends and significant others [24]; Muehlenkamp and colleagues [35] found that NSSI history was associated with the BPD symptom of interpersonal chaos, while suicide attempt history was associated with confusion about self. Although some researchers have reported no difference in suicidal ideation between the groups [37], others have found that those with a suicide attempt history report greater suicidal ideation [36], exposure to suicide attempts and death [24,36], and more repulsion with life [37]. Although additional research is necessary, findings suggest that attempted suicide may be associated with greater levels of psychiatric symptoms than NSSI. Future research should seek to elucidate the role of suicidal ideation in behaviors performed without suicidal intent, as research in adolescents has yielded mixed findings.

#### Risk factors for NSSI or attempted suicide

Of note, three longitudinal studies have investigated factors that uniquely predicted engagement in NSSI or attempted suicide among adolescents with MDD [38], treatment resistant depression [8], and nonclinical samples [39] (See Table 2). Wilkinson and colleagues [38] found that incidence of a suicide attempt within a 28-week follow up period was independently predicted by NSSI history and poor family functioning, while engagement in NSSI was independently predicted by NSSI history, hopelessness, presence of an anxiety disorder, female gender, and younger age. Importantly, the strongest predictor of either attempted suicide or NSSI during follow up was NSSI history at baseline [38]. Similarly, shorter time to a suicide attempt following treatment for depression was statistically predicted by NSSI history and hopelessness, while shorter time to engaging in NSSI was predicted by history of NSSI and physical and/or sexual abuse [8]. Only one study has investigated both risk and protective factors in common to and differentiating NSSI and attempted suicide. Wichstrøm [39] found that both NSSI and attempted suicide within a 5-year follow up period were associated with female gender, history of suicide attempts, and non-heterosexual sexual interest. However, NSSI during the follow up period was predicted by an NSSI history, and attempted suicide was predicted by suicidal ideation, an unstable self-concept, and conduct problems. It is important to note that unlike Wilkinson and colleagues [38] and Asarnow and colleagues [8], history of NSSI was not a risk factor for attempted suicide. Wichstrøm [39] also identified specific protective factors for each type of self-injurious behavior; satisfaction with social support uniquely protected against NSSI onset, and parental care uniquely protected against suicide attempt onset, suggesting that specific risk and protective factors may predict engagement in each type of self-injury.

**Table 2:** Summary of longitudinal studies investigating factors that uniquely predict engagement in NSSI or SA

Study	Sample	Risk Factor for NSSI	Risk Factor for SA
Asarnow et al. [8]	237 adolescents with treatment-resistant MDD	NSSI history Physical or sexual abuse history	NSSI history Hopelessness

Wichstrom [39]	2,924 high school students	*Risk Factors:*	*Risk Factors:*
		Female gender	Female gender
		History of suicide attempts	History of suicide attempts
		Non-heterosexual sexual interest	Non-heterosexual sexual interest
		Low Global self-worth	Low Global self-worth
		Sexual intercourse before age 15	Suicidal ideation
		NSSI History	Conduct problems
		*Protective Factors:*	*Protective Factors:*
		Satisfaction with social support	Parental Care

Wilkinson et al. [38]	164 treatment-seeking adolescents with MDD	NSSI history	NSSI history
		Hopelessness	Poor family functioning
		Presence of an anxiety disorder	High suicidality
		Female gender Younger age	

*Note*. NSSI = Non-suicidal self-injury. SA = Suicide attempt. MDD = Major depressive disorder.

#### NSSI as a risk factor for attempted suicide

Of particular importance given the prevalence of self-injurious behaviors, presence of an NSSI history or an attempted suicide history is associated with future self-injurious thoughts and behaviors in both adults and adolescents. Consistent with suicidal ideation as a risk factor for suicidal behavior, adolescents with a history of attempted suicide report greater suicidal ideation that those with a history of NSSI alone [12]. Adolescents with a history of both NSSI and attempted suicide also report greater suicidal ideation [6,12,26] and fewer reasons for living [6] than adolescents with an NSSI history alone.

History of suicidal behavior is a strong predictor of future suicidal behavior in adults and adolescents [4,40]. However, recent research suggests that a future suicide attempt is not only associated with self-injury with suicidal intent. History of NSSI has also been shown to predict attempted suicide among adolescents [38,41], and increased NSSI is associated with weaker suicidal ideation remission over time [42]. In fact, history of NSSI may be a stronger predictor of attempted suicide than a suicide attempt history in both adults [43] and adolescents [38]. Therefore, although NSSI is performed without suicidal intent by definition, incidents of NSSI-even without a co-occurring suicide attempt history-are of the utmost clinical importance as adolescents with NSSI alone are no less likely than those with histories of NSSI and attempted suicide to engage in self-injury [9], and previous NSSI behaviors have been demonstrated to be important predictors of future suicidal behaviors.

## Conclusions

To summarize, research has demonstrated that adolescents with a history of both attempted suicide and NSSI generally experience more severe symptomology than adolescents who have engaged in only one type of self-injury. However, because of the breadth of the variables assessed and the specific self-injury groups utilized in each study, definitive conclusions about factors that confer risk for engagement in both NSSI and attempted suicide cannot be drawn as of yet. Researchers have also begun to investigate risk factors specific to NSSI or attempted suicide. Attempted suicide at follow up is associated with poor family functioning [38], suicidal ideation, unstable self-concept, and conduct problems [39], and NSSI at follow up is associated with hopelessness, presence of an anxiety disorder, female gender, and younger age [38]. Although research suggests that suicidal ideation may be more strongly associated with suicide attempt history than NSSI history [6,12,26,36], NSSI is a significant predictor of subsequent NSSI and subsequent suicide attempts [38] and associated with a shorter duration without self-injurious behaviors following treatment [8].

Attempted suicide and NSSI commonly co-occur. However, the association between the behaviors is more complex as they may be associated with unique risk factors, and NSSI may be a risk factor for attempted suicide. This has important implications for clinicians and researchers. Mental health professionals must recognize the importance of identifying and treating NSSI. Although the behavior is associated with myriad negative consequences, a developing body of research suggests that NSSI may increase risk for attempted suicide, mandating early identification and treatment of adolescents who engage in the behavior, as well as the development of empirically supported prevention programs.

Further, research must continue to explore the associations between self-injury with and without suicidal intent. Longitudinal studies are necessary to determine the temporal relations between the behaviors, and researchers should systematically investigate the presence and role of established risk factors for one type of self-injury in the other. Research specifically investigating NSSI has identified difficulties in emotion regulation, negative life events including childhood abuse, and specific psychiatric symptoms and diagnoses such as BPD, depression, anxiety, and substance use as risk factors for the behavior [1,33]. Similarly, research specifically investigating attempted suicide has identified suicidal ideation, previous suicidal behaviors, interpersonal conflict, psychiatric disorders such as mood disorders, anxiety disorders, and substance abuse, parental psychopathology, family history of suicidal behavior, and environmental factors such as abuse and family relationships as risk factors [4,30-32]. By directly comparing these and other risk factors in both attempted suicide and NSSI, researchers will be able to establish the factors that differentiate NSSI from attempted suicide, as well as the factors common to both behaviors. Such research will allow for the continued development of an etiological model of self-injurious behaviors with and without suicidal intent, furthering our understanding of self-injury and our ability to intervene with these prevalent and serious behaviors.

## Abbreviations

NSSI: Non-suicidal Self-Injury; MDD: Major Depressive Disorder; PTSD: Post-traumatic Stress Disorder; BPD: Borderline Personality Disorder

## Competing interests

The authors declare that they have no competing interests.

## Authors' contributions

MA was responsible for the coordination of the manuscript. All authors participated in the literature review and contributed to the draft of the manuscript. All authors read and approved the final manuscript.

## Author's note

This research was supported in part by National Institute of Mental Health grant K23MH082824 awarded to Margaret S. Andover.
